# Dataset on the TIC-MOC cruise onboard the R/V Hespérides, March 2015, Brazil-Malvinas Confluence

**DOI:** 10.1016/j.dib.2018.12.004

**Published:** 2018-12-06

**Authors:** Dorleta Orúe-Echevarría, Josep L. Pelegrí, Iván J. Alonso-González, Verónica M. Benítez-Barrios, Patricia De La Fuente, Mikhail Emelianov, Marc Gasser, Carmen Herrero, Jordi Isern-Fontanet, Jesús Peña-Izquierdo, Sergio Ramírez-Garrido, Miquel Rosell-Fieschi, Joaquín Salvador, Martín Saraceno, Daniel Valla, Montserrat Vidal

**Affiliations:** a Departament d’Oceanografia Física i Tecnológica, Institut de Ciències del Mar, CSIC, Barcelona, Spain; b Oceomic, Marine Bio and Technology, S.L., Parque Tecnológico de Fuerteventura, Puerto del Rosario, Spain; c Centro de Investigaciones del Mar y la Atmósfera, UMI/IFAECI, CONICET, Buenos Aires, Argentina; d Departamento de Oceanografía, Servicio de Hidrografía Naval, DCAO/FCEN/UBA, Buenos Aires, Argentina; e Departament de Biologia Evolutiva, Ecologia i Ciències Ambientals, Universitat de Barcelona, Barcelona, Spain

## Abstract

This oceanographic dataset was gathered during the TIC-MOC cruise, which was designed to characterize the dynamics of the Brazil-Malvinas Confluence. The cruise was carried on board the R/V Hespérides, with departure from Ushuaia and arrival to Salvador de Bahía. A total of 66 conductivity-temperature-depth (CTD) stations were completed between 8 and 22 March 2015, offshore from the continental platform and within 45°S-35°S and 61°W-50°W. At each station, water samples were collected, which were used to calibrate the CTD salinity-oxygen sensors and to determine inorganic nutrient concentrations, and the horizontal current was measured. Along its track, the vessel recorded surface temperature and salinity, as well as the horizontal flow down to about 700 m. Lastly, eight position-transmitting drifters were launched and two profiling floats were deployed and later recovered.

**Specifications table**

**Table:** 

Subject area	*Oceanography*
More specific subject area	*Oceanographic cruise data*
Type of data	*Tables, figures, text files*
How data was acquired	*SeaBird 911 Plus multi-parametric probe, SBE-43 oxygen sensor, 12-l 24-Niskin-bottle rosette, 4-beam 300 kHz RDI Workhorse Monitor, 75 kHz Teledyne RDI VADCP, SBE 21 SeaCAT thermosalinograph, subsurface drifting buoys, Argo-type Apex profilers, AA3 HR Seal Analytical instrument*
Data format	*Analyzed, calibrated*
Experimental factors	*Quasi-synoptic oceanographic sampling of the Brazil Malvinas Confluence during 8–22 March 2015*
Experimental features	*Hydrographic, velocity and biogeochemical measurements were completed*
Data source location	*Offshore from the South American continental platform and within 45*°*S-35*°*S and 61*°*W-50*°*W.*
Data accessibility	*Data are with this article and are available at*http://dx.doi.org/10.17632/hk8t43z3t3.1
Related research article	*Orúe-Echevarría, D., Pelegrí, J.L., Machín, F., Hernández-Guerra, A., Emelianov, M. Inverse modeling the Brazil-Malvinas Confluence. Journal of Geophysical Research, accepted with minor revisions.**[8]*

**Value of the data**

• High resolution quasi-synoptic oceanographic data from an intensive survey at the Brazil-Malvinas Confluence on March 2015.
• Data can be used for the description of currents and property fluxes, for the characterization of the water masses, for process studies such as cross-frontal and vertical mixing, and for model validation and reanalysis.
• Data also includes Apex vertical profilers and drifting buoys which allows a Lagrangian description of the frontal system.

## 1 Data

This dataset presents the different measurements collected during an oceanographic cruise, with a careful description of the experimental design, instrument types, field methodology, data processing and sensor calibration. All data files are available at Mendeley Data (see the readme.txt file at http://dx.doi.org/10.17632/hk8t43z3t3.2 for a description of the actual contents of each data file).

**Fig. 1:**
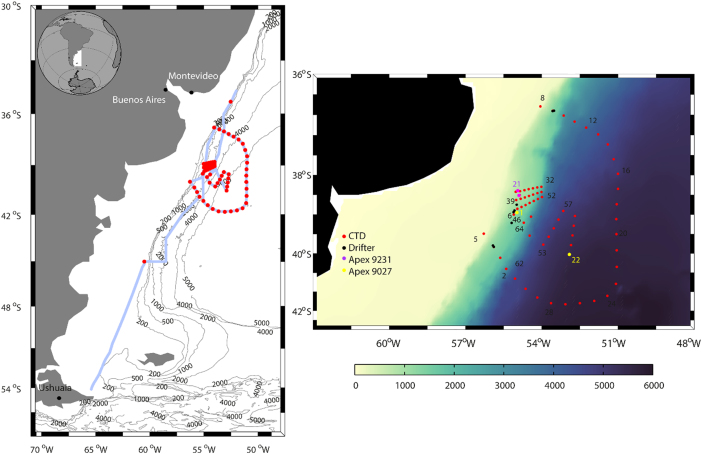
(Left) Vessel track during the TIC-MOC cruise (blue line) and location of the CTD stations (red dots). Black lines represent the 200, 500, 1000, 2000, 4000 and 5000 m isobaths. (Right) Map showing the location of all CTD stations (red dots), the position where the drifters were deployed (black dots), and the release and recovery locations for Apex profilers 9231 (magenta points) and 9027 (yellow points), on top the bathymetry (smoothed GEBCO 2008; color-coded in meters).

One type of data corresponds to the hydrographic stations (Fig. 1), a total of 66 stations including conductivity–temperature–depth (CTD) data, inorganic nutrient concentrations and water velocity obtained with a lowered acoustic Doppler current profiler (LADCP). In addition, it comprises flow velocity recorded along the ship track (with a vessel-mounted ADCP, VADCP) and near-surface temperature and salinity (with a thermosalinograph). The dataset also includes 42 vertical profiles completed by two profiling floats holding salinity, conductivity and pressure sensors (one of them having additional oxygen and fluorescence sensors) and the trajectories of eight near-surface drifters.

## 2 Experimental design, materials and methods

The TIC-MOC (Tipping Corners in the Meridional Overturning Circulation) cruise was carried onboard the R/V Hespérides (Spain), with departure from Ushuaia (Argentina) on 5 March 2015 and arrival to Salvador de Bahía (Brazil) on 30 March 2015 (Fig. 1). The study area was offshore from the continental platform, within 45°S–35°S and 61°W–50°W. Of the 66 completed hydrographic stations, 14 reached down to the seafloor, 24 stations down to 2000 m and 28 stations down to 400–500 m.

### 2.1 CTD and thermosalinograph data

At each hydrographic station (Fig. 1), CTD data were obtained with a SeaBird 911 Plus multi-parametric probe with redundant temperature and conductivity sensors and a SBE-43 dissolved oxygen sensor, Wetlabs AFL-NTU-RTD fluorescence and turbidity sensors and Biospherical QSP-2300 PAR (Photosynthetically Active Radiation) sensor. The CTD data were vertically averaged at 1 dbar pressure intervals after processing. The probe was mounted on a 12-l 24-Niskin-bottles rosette and water samples were collected at standard depths (Supplementary material) in all stations, which were later used for several biogeochemical analyses (only inorganic nutrients are presented in this article).

**Fig. 2:**
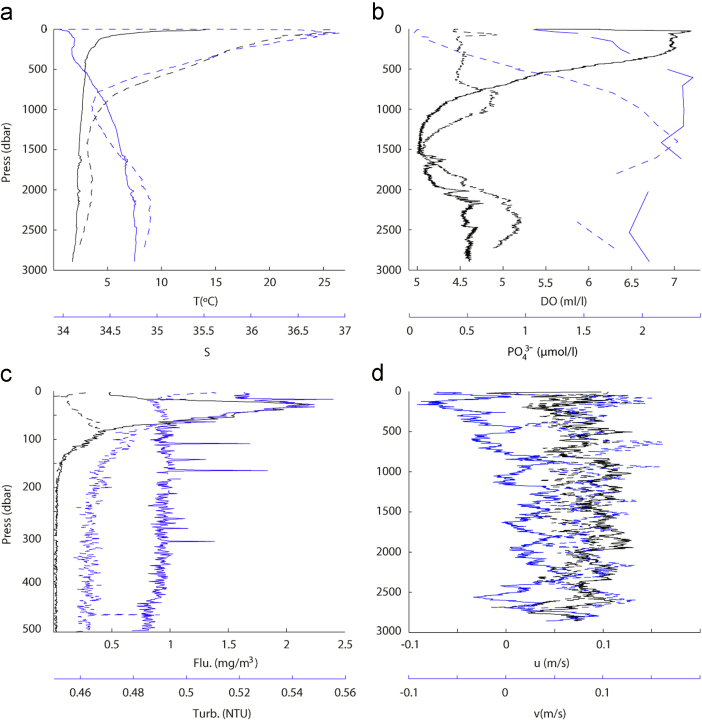
Vertical profiles for stations 2 (continuous line) and 10 (dotted line), respectively characterizing the subantarctic and subtropical waters, of (a) potential temperature (T in °C) and salinity (S), (b) dissolved oxygen concentration (DO in ml l^−1^) and phosphate concentration ($PO_{4}^{3-}$ in µmol l^−1^), (c) fluorescence (Flu in mg m^−3^) and turbidity (Turb in NTU), and (d) zonal (u in m s^−1^) and meridional (v in m s^−1^) velocity components. The black and blue lines correspond to the black and blue axes, respectively.

For each hydrographic station there are two sorts of files with CTD data: (1) the variables gathered in a near-continuous mode during the entire cast, with about three samplings per second, which are presented as 1-dbar averaged values, with separate files for the descending and ascending portions of the cast (hereafter CTD cast) (Fig. 2); (2) the variables gathered at the depth where the Niskin bottle was closed during the rosette ascension (hereafter bottle data).

**Fig. 3:**
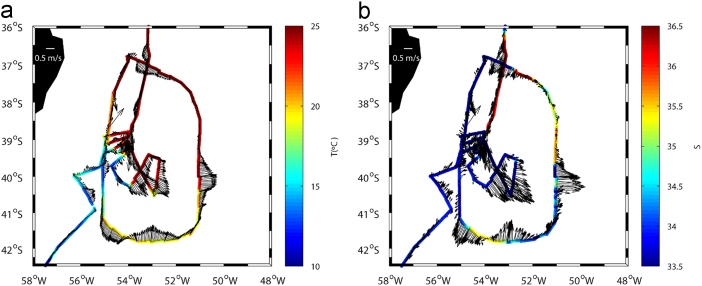
(a) Temperature (in °C) and (b) salinity as measured by the thermosalinograph (color-coded) along the ship׳s trajectory; the plot includes (a) the mean velocity in the top 700 m and (b) and the velocity at 20 m as obtained with the VADCP (vectors).

A SBE 21 SeaCAT thermosalinograph recorded temperature and salinity data in a continuous mode (one value every five seconds) from the vessel׳s underway system, approximately located at a depth of 5 m (Fig. 3). These data include the vessel׳s position, obtained through two differential global positioning systems.

### 2.2 LADCP and VADCP data analysis

Velocity data was recorded on each cast with the LADCP fixed to the rosette. It consisted of a dual-head setup (down-looking master, up-looking slave) four-beam RDI Workhorse Monitor with a working frequency of 300 kHz, set to obtain velocities in 4-m bins. Two configurations were initially prepared: one for casts reaching the sea bottom, which used staggered pings in order to avoid previous-ping interference, and another for profiles not reaching the sea bottom. During CTD profile 9 a major malfunction occurred, which caused that only the down-looking four-beam head worked properly on the next casts. A careful processing and data quality control confirmed that velocity profiles posterior to the instrument malfunction suffered no significant increase in observed error. LADCP data were processed with the Matlab LDEO IX toolbox [1], which uses CTD, vessel׳s navigation and bottom-tracking data.

The VADCP consists of an Ocean Surveyor Broadband/Narrowband 75 kHz Teledyne RDI equipment. The instrument was set to provide one velocity profile or ensemble every 5 min, between about 24 m and 800 m at 8-m bins. Raw data were quality controlled, corrected and edited with the Common Oceanographic Data Access System (CODAS) [2]. The Single-Ping processing scenario proposed by the CODAS software was used to process the VADCP data acquired with the RDI velocity processing software (VMDAS). The calibration of the instrument was checked and heading-corrected according to the bottom-tracking and water-tracking results.

### 2.3 Salinity

The CTD was equipped with duplicate conductivity sensors in order to detect any possible drift. In addition, a total of 65 water samples collected from the Niskin bottles were analyzed onboard using a Guildine Autosal 8400B salinometer (installed in a constant temperature room) with the objective of calibrating the CTD conductivity sensors. These water samples were gathered at stations scattered throughout the entire cruise, typically at depths where the vertical property gradients were low. Water samples were analyzed by lots, about 24 h after collection, after having equilibrated to laboratory temperature. Previous to each batch of samples, the instrument was calibrated using a standard seawater sample (SSS) from the International Association for the Physical Sciences of the Oceans. At the end of each batch, again a SSS was analyzed to verify the instrument׳s stability.

**Fig. 4:**
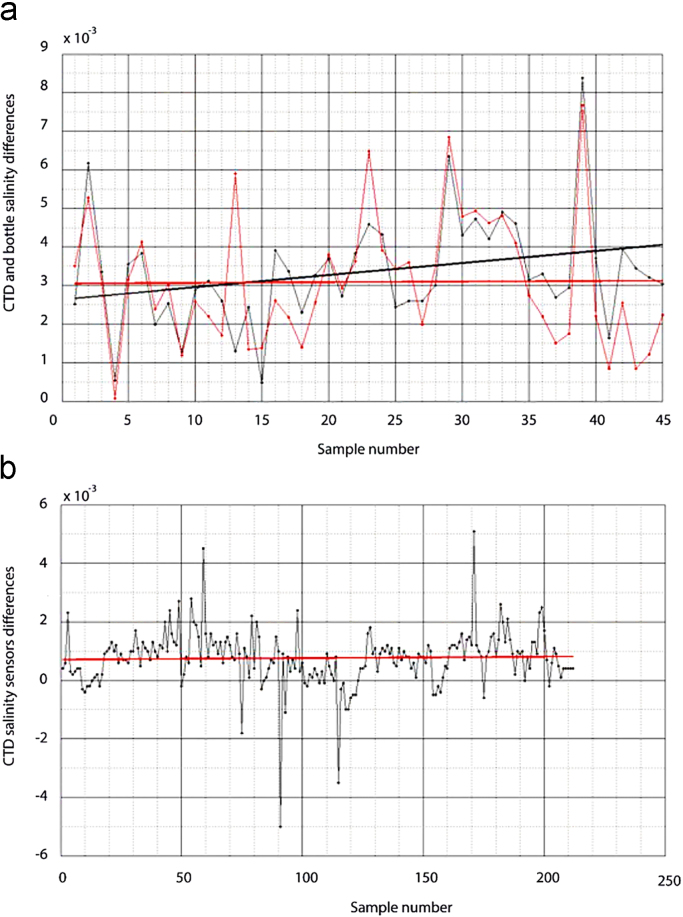
(a) Differences between the CTD salinity data (thin lines) and the salinometer measurements, and linear adjustments for these differences after discarding outliers (thick lines). The black and red lines correspond to the primary and secondary sensors, respectively. (b) Difference between the CTD primary and secondary salinity sensors in bottles closed deeper than 1000 m. The red line shows the linear adjustment.

The comparison between the salinity measured with the salinometer and the salinity derived from the CTD shows a mean offset of 0.003 and a shift of 0.0014 for the primary CTD conductivity sensor and a mean offset of 0.003 and a variation of 7 × 10^−5^ for the secondary sensor, with the shift corresponding to the 15-day entire cruise period (Fig. 4a). Further, a comparison of the primary and secondary CTD conductivity sensors for all bottles closing at depths higher than 1000 m shows an offset of 7.1×10^−4^, which is less than the differences with the salinometer (Fig. 4b). Therefore, the measurements from both CTD conductivity sensors were stable throughout the cruise, particularly the secondary one that had an offset close to the salinometer detection limit.

### 2.4 Oxygen sensor calibration

**Fig. 5:**
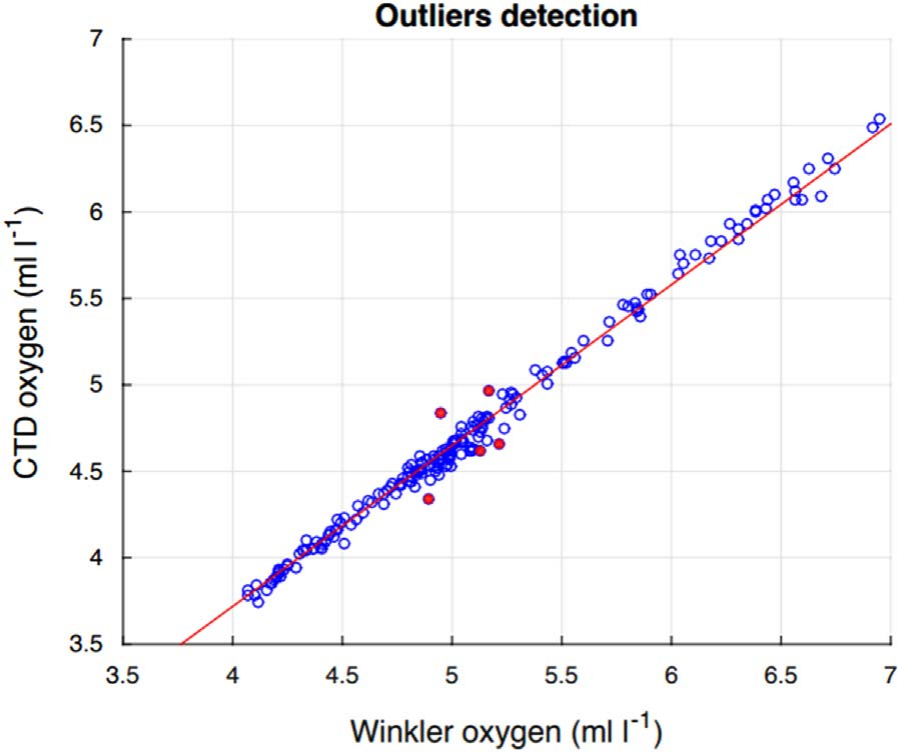
Linear regression between the Winkler and CTD oxygen concentrations for the samples used to compute the correction factor. The red dots indicate outliers eliminated for the calibration.

In order to calibrate the CTD oxygen sensor [3], 183 water samples from the Niskin bottles were used to determine the dissolved oxygen concentration via the Winkler-titration method [4]. The comparison shows good agreement between the CTD and Winkler method oxygen measurements (Fig. 5). The correction factor for the CTD oxygen is 1.0746, with no specific temporal or spatial significant dependences. After using the correction factor, the difference between CTD and Winkler oxygen concentrations is 0.002 ± 0.051 ml l^−1^. This factor has been applied to the enclosed datasets.

### 2.5 Drifters

**Fig. 6:**
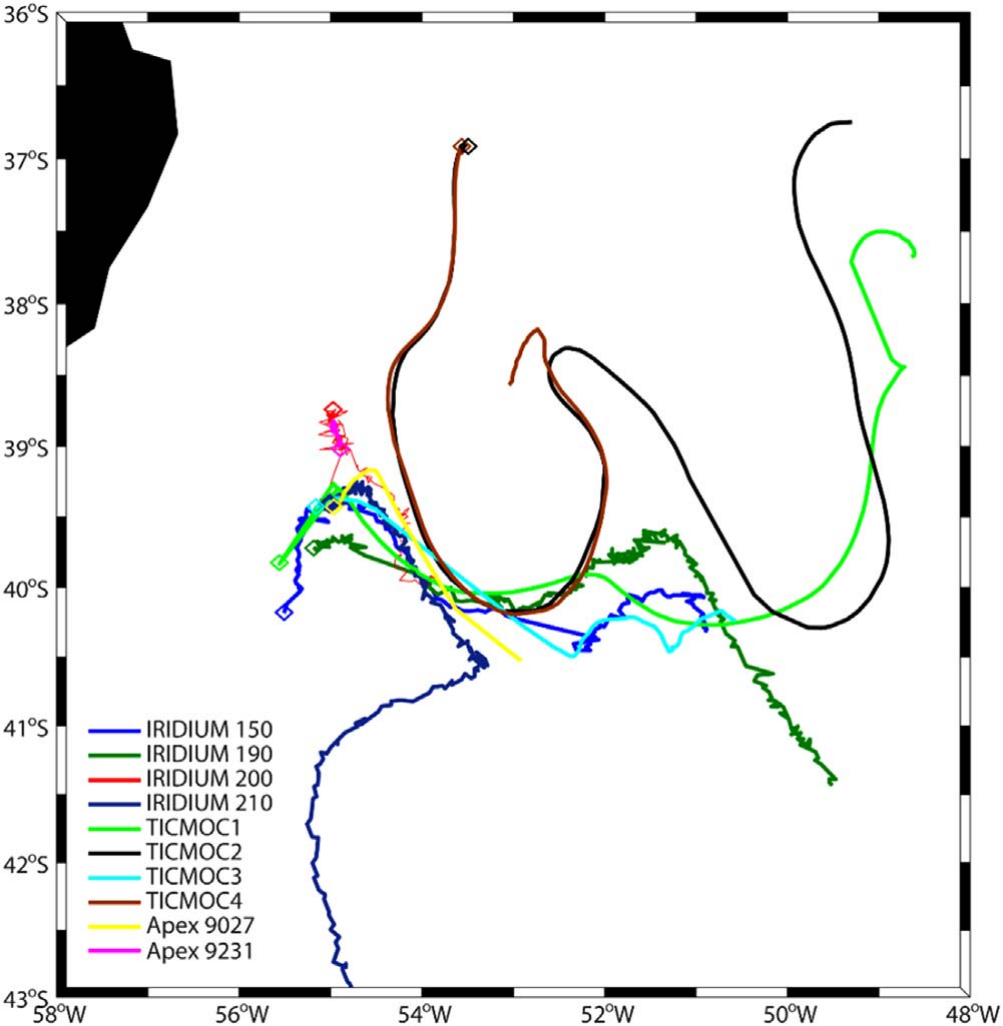
Trajectories of the surface drifters and profiling floats between their launching and 26 March 2015, with the deployment point indicated by a diamond.

Eight subsurface drifting buoys (drifters) were launched during the cruise (Fig. 1, Fig. 6, Table 1). These drifters have a spherical surface buoy, containing the batteries and the electronics of the system, and a 15-m holey sock dragged at 100–200 m depth [5]. The electronics consists of a global positioning system and a satellite data transmitter (Global Star in four buoys and Iridium in the other four); positions were acquired every 30 min in all drifters. The data presented corresponds to the period between the launching and the 26 March 2015.

**Table 1:** Deployment times and positions for the near-surface drifters.

Name	Date (yyyy/mm/dd)	Time (GMT)	Latitude S	Longitude W	Transmitter	Drogue depth (m)
TICMOC 1	2015/03/11	08:20:00	39^o^ 14.85´	54^o^ 58.90´	Global Star	100
TICMOC 2	2015/03/12	08:24:00	36^o^ 53.93´	53^o^ 31.11´	Global Star	100
TICMOC 3	2015/03/10	16:50:00	40^o^ 19.80´	55^o^ 52.80´	Global Star	200
TICMOC 4	2015/03/12	08:08:00	36^o^ 54.39´	53^o^ 34.23´	Global Star	200
IRIDIUM 150	2015/03/10	17:31:00	40^o^ 18.45´	55^o^ 53.18´	IRIDIUM	100
IRIDIUM 190	2015/03/11	03:50:00	39^o^ 44.75´	55^o^ 12.35´	IRIDIUM	100
IRIDIUM 200	2015/03/11	06:34:00	39^o^ 29.50´	55^o^ 05.50´	IRIDIUM	100
IRIDIUM 210	2015/03/11	11:21:00	39^o^ 00.29´	54^o^ 52.25´	IRIDIUM	100

### 2.6 Vertical profiling floats

Two Argo-type Apex profilers (Teledyne Webb Research) were deployed and recovered during the cruise (Fig. 1, Fig. 6, Table 2). Both floats were equipped with SBE41CP CTD sensors (float 9027 also had fluorescence and oxygen sensors) and Iridium transmitters with bidirectional communication. These transmitters allowed modifying the programmed cycle while the drifter was in the water, changing the profiling and parking depths and the time the float remained at the parking depth and at the sea surface. In all cases (except for float 9027 between profiles 20 and 21) the profilers were asked to not remain at the parking depth and to stay at the sea-surface only until it successfully connected with the satellite.

**Table 2:** Deployment and recovery times and positions for the profiling floats.

Float	Start			End		
	Date (yyyy/mm/dd)	Latitude S	Longitude W	Date (yyyy/mm/dd)	Latitude S	Longitude W
9027	2015/03/11	39^o^ 30.01´	55^o^ 06.00´	2015/03/19	40^o^ 31.50´	52^o^ 57.78´
9231	2015/03/11	39^o^ 00.29´	54^o^ 52.25´	2015/03/17	38^o^ 53.52´	54^o^ 56.52´

Float 9027 was deployed on 11 March and recovered on 19 March, completing a total of 22 profiles. The maximum pressure was about 500 dbar for the first 12 profiles and around 750 dbar for the remaining 10 profiles. The first 13 profiles sampled in continuous mode (one measurement approximately every 2 dbar) and since profile 14 the vertical resolution was set to 10 dbar. After profile 17, the communication with the float was lost, emerging two days later after having performed three profiles, two of them (18 and 19) not positioned. This was caused by the presence of significantly fresh Rio de la Plata waters in the uppermost 10–20 m of the water column (e.g. at 04:04:56 UTM on 16 March, with the buoyancy bladder fully extended, drifter 9027 was at 11.5 dbar; having no capacity to gain further buoyancy, it descended again to the parking depth). Between profiles 20 and 21 the float remained at the sea surface while providing 33 surface positions.

Float 9231 was deployed on 11 March and recovered on 17 March after doing 20 profiles. The first 9 profiles reached down until around 300 dbar and from profile 10 to 20 the maximum pressure was about 800 dbar. During the first 14 casts, continuous-mode sampling was set for the upper 200 dbar and 10-dbar sampling further deep; for the last 6 profiles continuous-mode sampling was set for the entire profile.

### 2.7 Inorganic nutrients

**Fig. 7:**
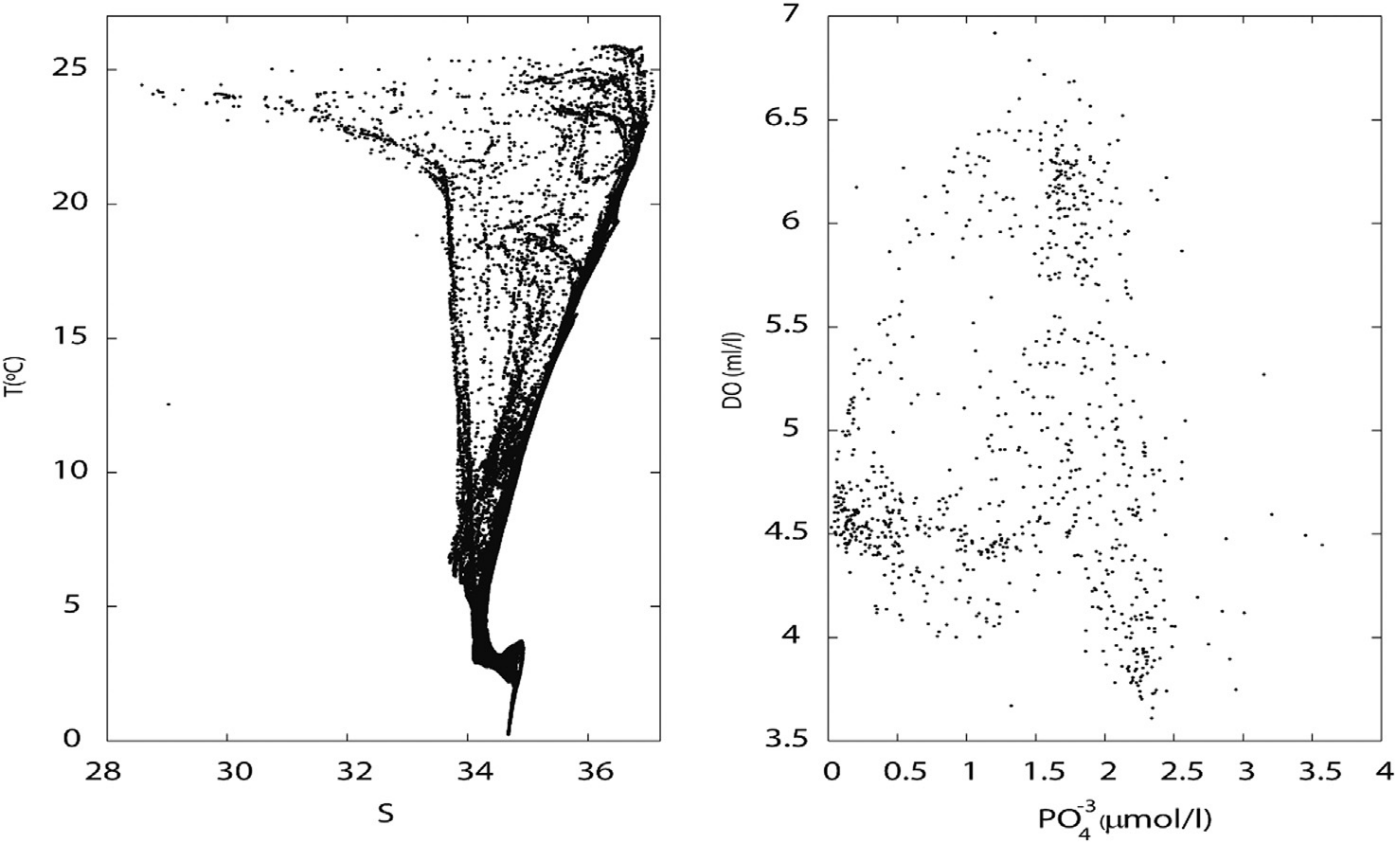
(Left) Potential temperature - salinity (T-S) and (right) dissolved oxygen - phosphate concentration ($DO-PO_{4}^{3-}$) diagrams.

At each station, 50-ml water samples were obtained from the Niskin bottles and later used to determine inorganic nutrient concentrations (nitrate, nitrite, silicate and phosphate). These water samples correspond to the standard water depths (Supplementary materials) plus a selected number of depths, which changed depending on the maximum sampling depth and the observation of particular features during the descending CTD cast. Samples were immediately frozen at −20 °C and analyzed within three months at the Institute of Marine Sciences in Barcelona using an AA3 HR Seal Analytical instrument. The nutrients analyses allowed determining the concentrations of nitrate, nitrite, phosphate and silicate [6]. Data detection limits and accuracies are included in Table 3 [7]. (Fig. 7).

**Table 3:** Lowest range (µM), coefficient of variation and detection limit (µM) in the lowest range of the inorganic nutrient analysis method.

	Lowest range	Coefficient of variation (10 replicates at 50%)	Detection limit in lowest range (MDL)
Nitrate + Nitrite	0 to 2.9	0.21%	0.0100
Nitrite	0 to 0.3	0.20%	0.0015
Silicate	0 to 8.0	0.50%	0.0160
Phosphate	0 to 6.5	0.20%	0.0200
