# Supplementary material for: Dataset on the TIC-MOC cruise onboard the R/V Hespérides, March 2015, Brazil-Malvinas Confluence
Source: Data Brief. 2018 Dec 6;22:185–94. doi: 10.1016/j.dib.2018.12.004 (PMC6301983; doi:10.1016/j.dib.2018.12.004)
Supplement: Supplementary file 2 — Supplementary material [file mmc2.docx]

**Supplementary Materials**

Dataset on the TIC-MOC cruise onboard the R/V Hespérides, March 2015,

Brazil-Malvinas Confluence

Dorleta Orúe-Echevarría, Josep L. Pelegrí, Iván J. Alonso-González, Verónica M. Benítez-Barrios, Patricia De La Fuente, Mikhail Emelianov, Marc Gasser, Carmen Herrero, Jordi Isern-Fontanet, Jesús Peña-Izquierdo, Sergio Ramírez-Garrido, Miquel Rosell-Fieschi, Joaquín Salvador,

Martín Saraceno, Daniel Valla, Montserrat Vidal

The Supplementary Materials contain one single table which follows next.

Table S1. Water sample depths for (left column) bottom-reaching and (right column) 2000-m depth stations

| *Bottom-reaching (m)* | *2000-m depth (m)* |
| --- | --- |
| *Bottom* | *2000* |
| *5500* | *1800* |
| *5000* | *1600* |
| *4500* | *1400* |
| *4000* | *1200* |
| *3500* | *1000* |
| *3000* | *900* |
| *2500* | *800* |
| *2000* | *700* |
| *1750* | *600* |
| *1500* | *500* |
| *1250* | *450* |
| *1000* | *400* |
| *800* | *350* |
| *600* | *300* |
| *500* | *250* |
| *400* | *200* |
| *300* | *150* |
| *200* | *100* |
| *150* | *75* |
| *100* | *Depth of deep chlorophyll maximum* |
| *Depth of deep chlorophyll maximum* | *50* |
| *50* | *25* |
| *5* | *5* |
